# Leptin Signaling in Liver Tissue of a Transgenic Breast Cancer Mouse Model

**DOI:** 10.7759/cureus.6737

**Published:** 2020-01-22

**Authors:** Bilge Guvenc Tuna, Margot P Cleary, Pinar B Demirel, Soner Dogan

**Affiliations:** 1 Biophysics, Yeditepe University Faculty of Medicine, İstanbul, TUR; 2 Food Science and Nutrition, University of Minnesota Hormel Institute, Austin, USA; 3 Medical Biology and Genetics, Maltepe University Facullty of Medicine, Istanbul, TUR; 4 Medical Biology, Yeditepe University School of Medicine, Istanbul, TUR

**Keywords:** leptin, leptin receptor, mammary tumor, breast cancer, liver, transgenic mouse model

## Abstract

Leptin, an adipocytokine, is secreted from various tissues including the liver. The roles of both leptin and leptin receptor (ObR) in numerous pathophysiological conditions including mammary tumor (MT) development have been reported. However, the roles of leptin signaling-related proteins in the liver have not been reported previously in MT development. The objective of this study was to examine the expression levels of leptin and ObR in liver tissue of a transgenic breast cancer mouse model to investigate whether the roles of leptin in MT development are systemic or local. MMTV-TGF-α transgenic female mice were fed ad-libitum from week 10 up to week 74. Protein expression levels of leptin and ObR were measured in liver tissues of 74-week-old MMTV-TGF-α mice with and without MT by western blot. Serum leptin and insulin levels were measured using a enzyme-linked immunosorbent assay. Protein expression levels of leptin and ObR were similar in mice with MT compared to the ones without MT. Serum leptin and insulin levels were also not significantly different between the two groups. These results indicate that the effects of leptin signaling in MT development might be important at a local tissue level, such as mammary fat pad, and not as important at a systemic level.

## Introduction

Leptin is a 16-kDa adipokine secreted primarily from the adipose tissue [1]. Leptin and its receptors have been reported to have roles in many physiological events mostly related to food intake, energy consumption, hemostasis, ovulation, fertilization, angiogenesis, obesity and breast cancer in a variety of species including mice and humans [2]. Additionally, increased expression of leptin and its receptors were demonstrated in breast cancer cell lines as well as in human breast cancer tissues [3,4].

Leptin acts by binding its transmembrane receptors, of which six subtypes have been reported so far [5]. Expression of leptin and its receptors have been reported in different types of cells and tissues such as adipose, liver, lung, ovary and breast cancer [6-9]. Although there are several studies examining the association between serum leptin levels and cancer development, there is a comparably limited number of studies investigating the role of leptin receptors in cancer development. Previous studies from our laboratory reported that leptin and leptin receptor-deficient mice do not develop mammary tumors (MTs): when Lep strain mice were crossbred with transgenic MMTV-TGF-a mice, obese MMTV-TGF-α/Lep(ob)Lep(ob) mice did not develop oncogene-induced MTs, although their lean littermates did [10]. Besides, Lepr mice that exhibit a mutation in the leptin receptor were crossbred with MMTV-TGF-α mice, and again no MTs were detected in obese MMTV-TGF-α/Lepr(db)Lepr(db) mice [11]. One could argue that MMTV-TGF-α MTs are not affected by obesity; however, we also showed that MMTV-TGF-α mice with diet-induced obesity had shortened MT latency [11]. Thus, MT development was observed in the presence of an active leptin signaling. These findings led us to hypothesize that leptin is a growth factor for breast/mammary cancer cells.

Insulin is a hormone that regulates glucose levels in blood. High insulin levels were reported in obese patients, and obesity is known to be associated with many cancer types including breast cancer. Serum insulin levels are increased in nondiabetic overweight breast cancer patients, and this is a risk factor for breast cancer development [12]. Indeed, insulin administration has been reported to increase the MT incidence rate in a chemically induced MT rat model [13]. Studies investigating the association between serum leptin levels and breast cancer revealed conflicting results: some studies reported a positive correlation between serum leptin levels and breast cancer risk, while others found either no association or a negative association [14-18].

The association between leptin signaling and breast cancer development has been previously examined. However, the relation between the leptin signaling related proteins in liver and MT development has not been reported, which may be important to understand whether the leptin signaling is associated with MT development at a systemic or local level. In this study, protein expression levels of both leptin and leptin receptor (ObR) in liver tissue were measured and compared in 74-week-old MMTV-TGFa mice with and without MT development. Serum leptin and insulin levels in these mice were also examined.

## Materials and methods

Materials

Primary antibodies against leptin and ObR and the secondary antibody alkaline phosphatase-conjugated anti-rabbit IgG were purchased from Santa Cruz Biotechnology Inc. (Santa Cruz, CA). β-actin antibody was purchased from Delta Biolabs (Vandell Way Campbell, CA). Enhanced chemifluorescence (ECF substrate) was obtained from Amersham Biosciences (Piscataway, NJ). Tris-base solution (TBS), Tris/Glycine/SDS buffer and polyacrylamide gradient gels were purchased from Bio-Rad Laboratories (Hercules, CA). Polyvinylidene difluoride (PVDF) membranes were purchased from Immobilon-P, Millipore (Billerica, MA). Protein extraction kits were purchased from Pierce Corp (Rockford, IL). Proteinase inhibitors were purchased from G-Biosciences/Genotech (St. Louis, MO). The mouse diet was purchased from Harlan Teklad (Madison, WI).

Mice and study design

MMTV-TGF-α (C57BL/6) female mice were used. This transgenic mouse strain was originally developed in the laboratory of Dr. Robert J. Coffey [19]. These mice overexpress human TGF-α, a growth factor overexpressed in breast cancer. The mice were obtained from a breeding colony maintained at the Hormel Institute University of Minnesota using the breeding protocol and genotyping assay previously described [20]. Mice were fed AIN-93M diet, which was supplied from Harlan Teklad from 10 to 74 weeks of age. Each mouse was individually caged. Mice were observed and palpated to identify the presence of MTs or other health problems weekly. At 74 weeks of age, blood samples were obtained from all mice at euthanasia. Liver samples, MTs and mammary fad pad tissues were collected gently. In addition, mammary tissues and retroperitoneal and parametrial fat pads which were located around periphery of kidneys and visceral tissues, respectively, were removed. A portion of each tissue sample was preserved in formalin and sent to the Department of Pathology and Laboratory Medicine (Mayo Foundation, Rochester, MN) for histopathological analyses to determine the malignancy status in a blinded fashion. All confirmed MT samples were histopathological grade 2. The remaining tissues and serum samples were stored at -80°C until used for future analyses. According to the MT status, mice were divided into two groups, those with (MT-positive) and those without (MT-negative) tumors. All procedures with mice were done under the guidelines and approval of the University of Minnesota Institutional Animal Care and Use Committee in an AAALAC accredited facility.

Western blot analysis of leptin and leptin receptor in liver

Tissue samples from individual mice were homogenized in extraction buffer with protease inhibitors. Total protein was extracted using a T-PER Tissue Total Protein Extraction reagent as described in the manufacturer’s protocol (Pierce, Rockford, IL) and quantitated using the Bio-Rad protein assay kit with bovine serum albumin as the standard (Bio-Rad Laboratories, Hercules, CA). Extracted proteins were electrophoresed on a 4%-15% polyacrylamide gradient gel and then transferred to a PVDF membrane. Blots were blocked in TBS containing 1% milk concentrate and 0.1% Tween-20. The PVDF membranes were incubated with appropriate primary antibodies against leptin, ObR and b-actin proteins in the liver samples. Consequently, membranes were incubated with a secondary antibody conjugated to alkaline phosphatase. ECF substrate was used to visualize the bands using a Storm 840 Machine Imaging System (Amersham Biosciences, Piscataway, NJ). Standard molecular weight markers were run simultaneously for comparing molecular weights of the visualized proteins. The intensity of western blot bands was quantified by densitometric analysis using the program UN-SCAN-IT gel (Silk Scientific, Orem, UT). Results were expressed as the ratio of intensity of the protein interest to that of b-actin from the same sample. Samples from eight different animals were used for the western blot analysis. 

Measurement of serum leptin and insulin levels by enzyme-linked immunosorbent assay

Blood samples were collected following euthanasia, five hours after mice were given their daily allotment of food. Leptin and insulin levels in serum were measured using a commercially available Mouse Adipokine LINCOplex Kit 96-Well Plate Assay (Linco Research, St. Charles, MO). Results were read on the Luminex 100 instrument. For the correlation and ratio metric analysis, the values from individual animals were used.

Statistical analysis

**Figure 1:**
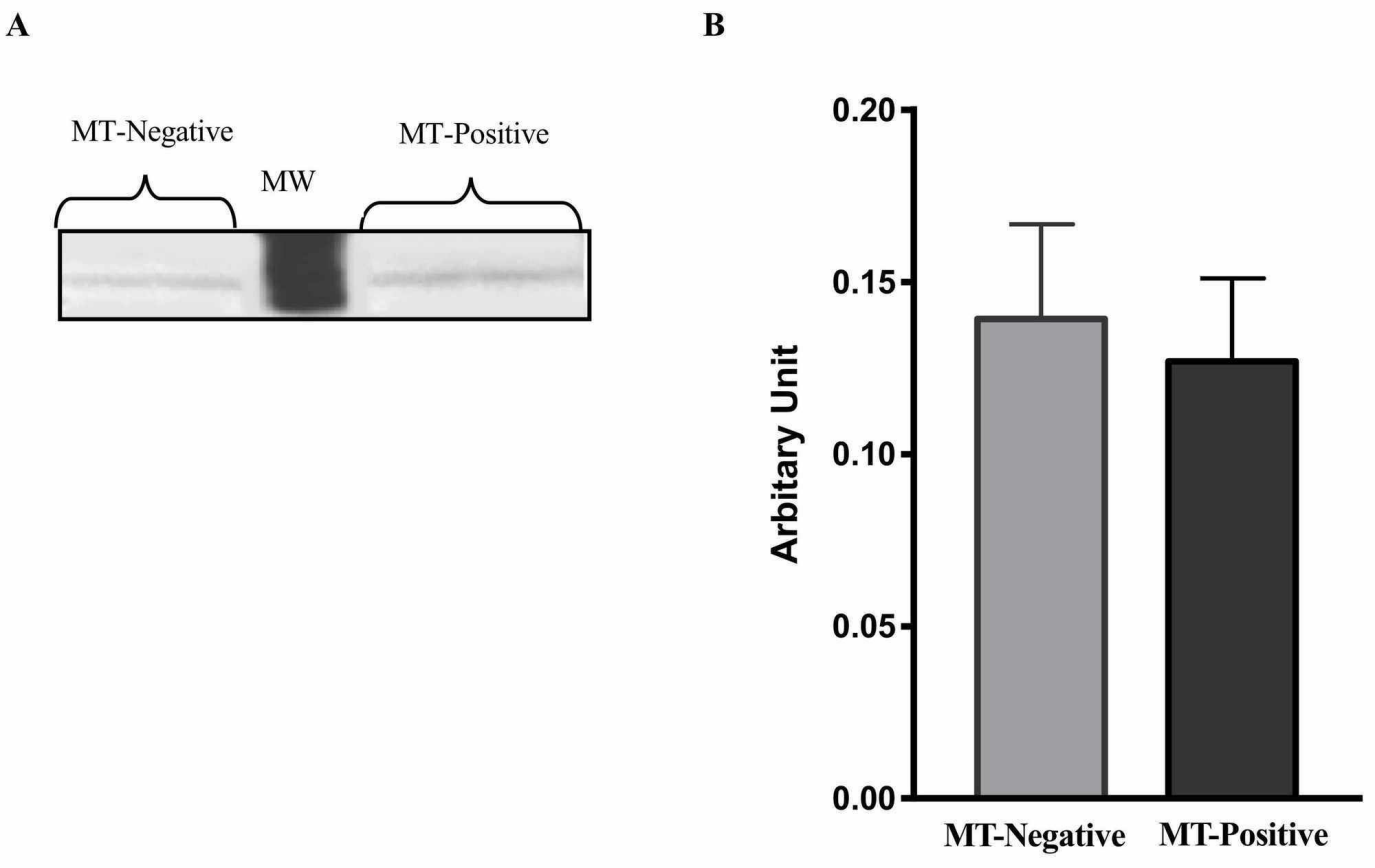
Leptin protein expression levels in liver tissue of mice with and without mammary tumor (MT) development at 74 weeks. (A) Western blot analysis of leptin levels in liver tissue samples of MT-negative and MT-positive mice (n=8). (B) Average density values for leptin levels in MT-negative and MT-positive mice (n=8). Data represent standard error of the mean. MW, molecular weight marker.

Data are presented as standard error of the mean (SEM). Student’s t test was used to determine whether the differences between two groups were statistically significant. Statistical significance at P<0.05 is indicated by *. “n” refers to number of individual mice in each group and “n” is eight unless otherwise is indicated in the legend of Figure 1.

## Results

MT detection

In these transgenic mice (MMTV-TGFalfa, C57Bl/6), the MTs were developed at the interscapular region of the body and axillary areas. Body compositions of the two groups, MT-positive and MT-negative, were similar in body weight and fat amount. There were no significant differences between MT-positive and MT-negative mice with respect to body weight, parametrial, retroperitoneal, mammary fat pad and total fat pad weights.

Leptin and leptin receptor (ObR) protein expression levels in liver tissue

Leptin protein expression levels were measured in liver samples taken from MT-positive and MT-negative mice. Leptin protein expression levels in liver tissue samples taken from mice developed MT were similar compared to the mice that did not develop MT (Figure 1, P>0.05).

**Figure 2:**
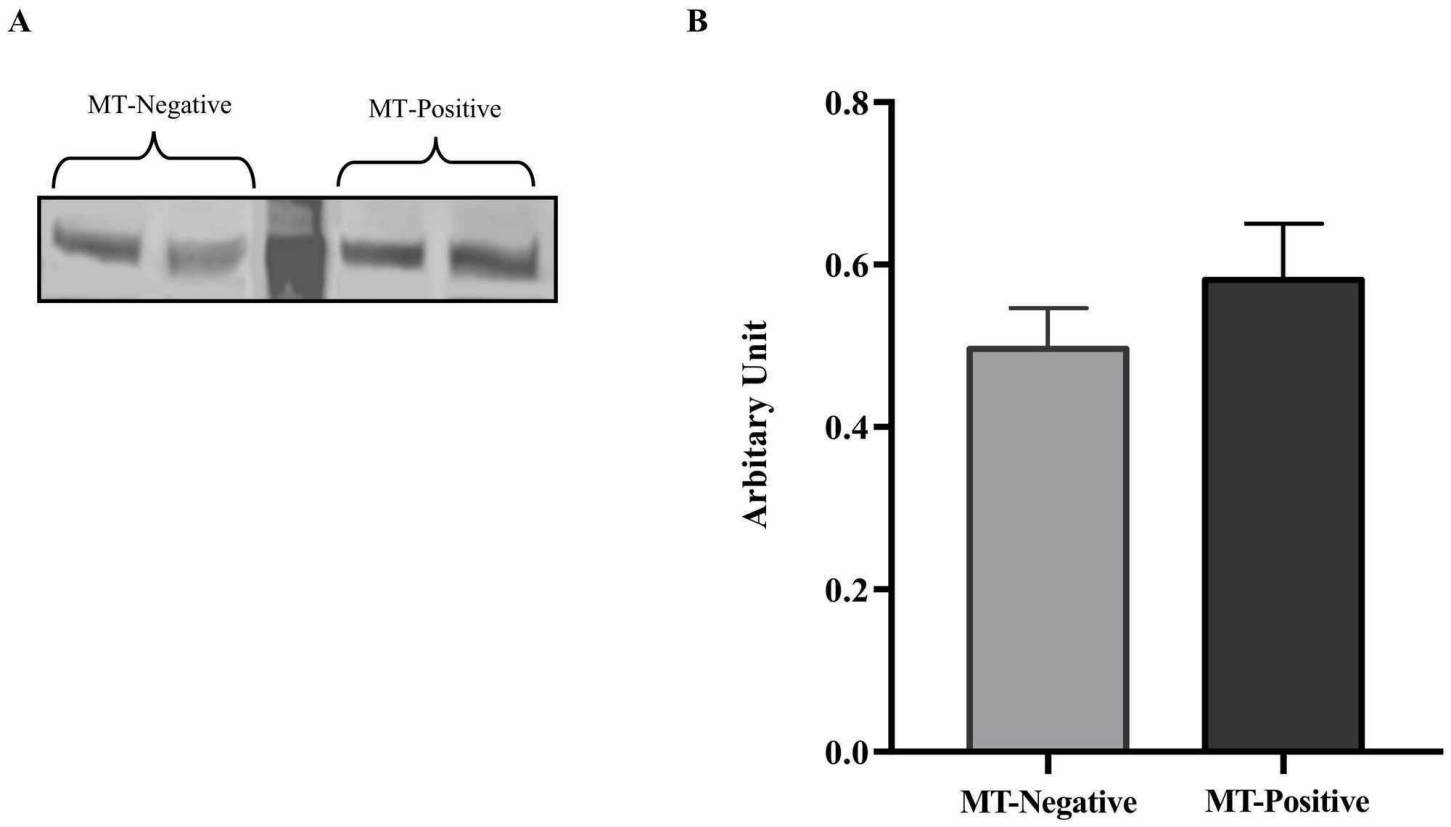
Leptin receptor (ObR) protein expression levels in liver tissue of mice with and without mammary tumor (MT) development at 74 weeks. (A) Western blot analysis of leptin receptor (ObR) protein levels in liver tissue samples of MT-negative and MT-positive mice (n=8). (B) Average density values for ObR levels in MT-negative and MT-positive mice (n=8) with standard error of the mean. MW, molecular weight marker.

In addition, protein expression levels of ObR were also not significantly different between liver samples taken from MT-positive and MT-negative mice (Figure 2, P>0.05). 

Leptin levels in serum

**Figure 3:**
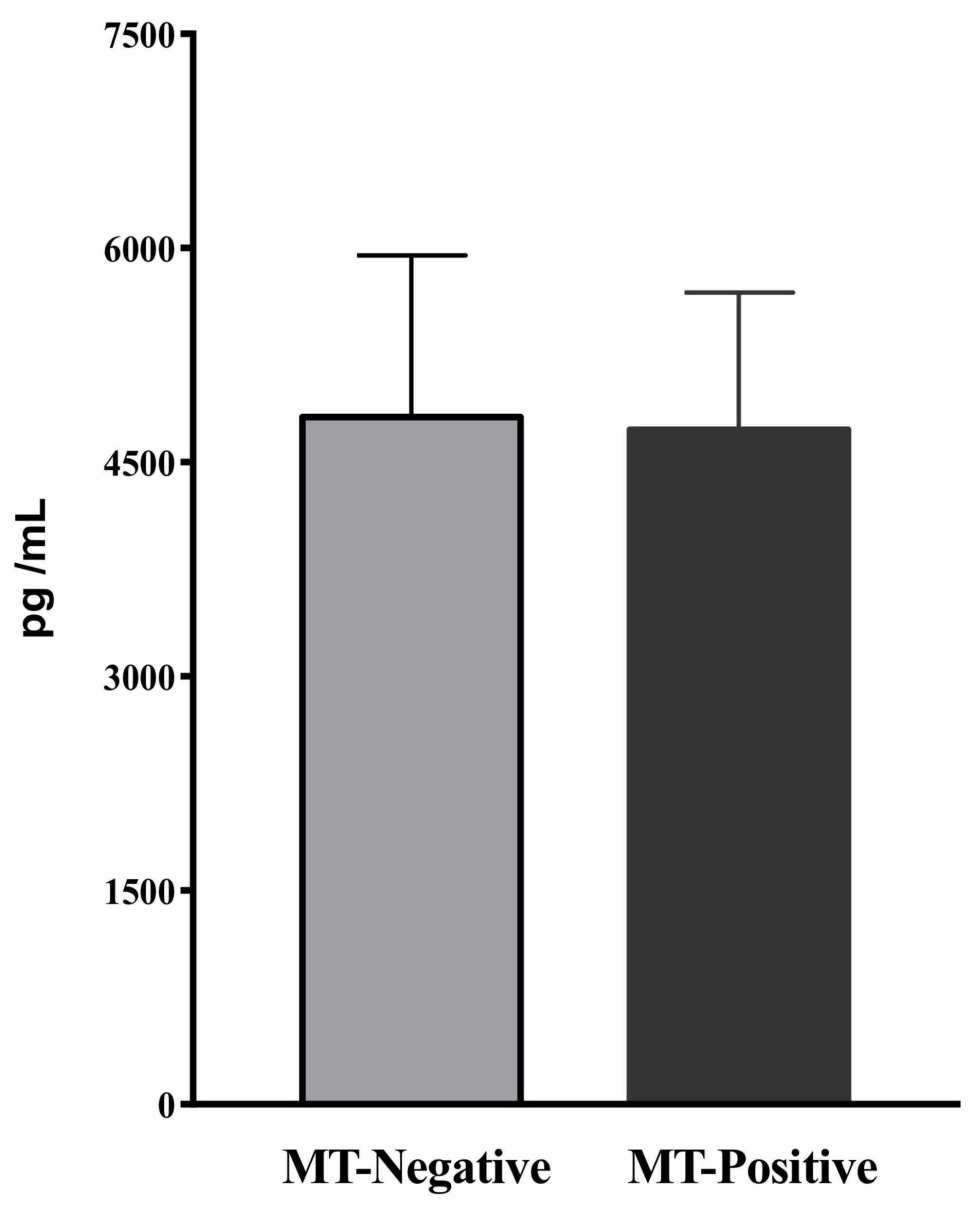
Serum leptin levels in mice with and without mammary tumor (MT) development at 74 weeks by ELISA. Data are the average serum leptin levels of individual MT-negative and MT-positive mice (n=8) with standard error of the mean. ELISA, enzyme-linked immunosorbent assay.

In order to study the relationship between leptin protein expression levels and serum leptin levels in the transgenic breast cancer mouse model, serum leptin levels were also measured. There were no significant differences between the MT-positive and MT-negative groups in serum leptin levels at the terminal age, 74 weeks (Figure 3). The average serum leptin levels at week 74 for MT-positive and MT-negative mice were 4.73±0.962 and 4.82±1.133 ng/mL, respectively (Figure 3, P>0.05). 

Serum insulin levels

**Figure 4:**
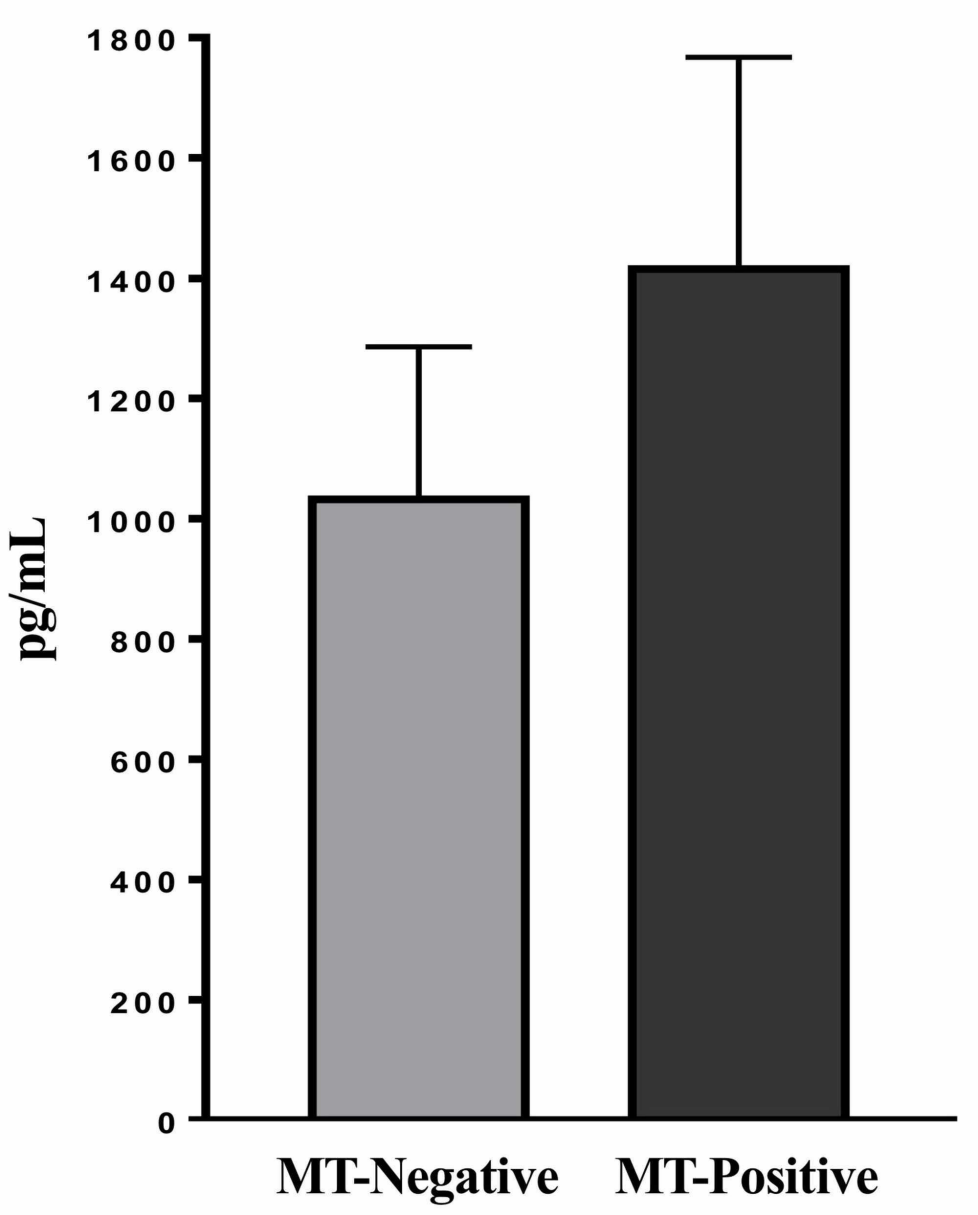
Serum insulin levels in mice with and without mammary tumor (MT) development at 74 weeks by enzyme-linked immunosorbent assay (ELISA). Data are the average serum insulin levels of individual MT-negative and MT-positive mice (n=8) with standard error of the mean.

Serum insulin levels were measured to determine whether MT development affects insulin levels in serum. There was no significant difference in serum insulin levels between MT-positive and MT-negative mice at 74 weeks (Figure 4). The average serum insulin levels at week 74 for MT-positive and MT-negative mice were 1.03±0.25 and 1.41±0.35 ng/mL, respectively (P>0.05).

## Discussion

Increased expression of leptin and its receptors was demonstrated in breast cancer cell lines and in human breast cancer tissues, since leptin and its receptors play important roles in apoptosis, cell proliferation and differentiation, mTOR (mammalian target of rapamycin) signaling, glucose regulation, inflammation, neurogenesis and gynecological diseases [3,4,21-23]. Majority of the studies investigating the role of leptin and its receptors in breast cancer development were conducted with serum samples, mammary fat pad tissue or MT samples [21-24]. However, to the best of our knowledge, there is no previous study examining the association between the leptin and leptin receptor expression in liver and MT development. Determining the expression of leptin and leptin receptors in liver tissue in the presence of MT is important to understand whether the leptin signaling is associated with MT development at a systemic or local level. The reasons of choosing the liver tissue were because leptin secretion occurs in liver tissue and it has a homogenous organ structure in mice with or without MT development.

Our data showed that the expression levels of leptin and ObR were similar in liver tissue of mice at 74 weeks of age with or without MT development. Previous studies conducted in local tissues (mammary fat pad, MT and adjacent healthy tissue) revealed a significant upregulation in leptin signaling judged by the increased expression levels of leptin and its receptor. In this context, Liang et al. demonstrated significantly higher leptin mRNA levels in MT tissues compared to the adjacent healthy tissues of breast cancer patients [25]. Expression of ObR was also reported to be increased in the majority of the breast ductal carcinoma cells compared to normal mammary epithelial cells by immunohistochemistry [15]. Similarly, Garofalo et al. showed that leptin and ObR protein expression levels were significantly higher in primary and metastatic breast cancer compared to noncancerous tissues. They also showed that expression levels of both leptin and ObR were higher in grade 3 MTs compared to lower grade MTs [26]. These results together with ours indicate that the association between leptin signaling and MT development is local rather than systemic, since previous studies showed enhancement of leptin signaling in MT, while we did not determine any significant difference in liver tissue in the presence of MT.

Current data regarding to the association between serum leptin levels and MT development are controversial. Some studies suggested high levels of serum leptin as a biomarker for breast cancer development [27,28]. Since we determined no significant difference in serum leptin levels between MT-positive and MT-negative mice, our results are in accordance with the previous studies reporting no correlation between serum leptin levels and breast cancer development [14,16,25].

Effects of insulin on cell proliferation and tumor development have also been reported in cell culture as well as animal studies [13]. There are studies reporting higher serum insulin levels in breast cancer patients compared to the healthy control [12]. On the other hand, others demonstrated no significant difference in serum insulin levels between breast cancer patients and healthy individuals [29]. In this study, we also found no significant difference in serum insulin levels between MT-negative and MT-positive mice at 74 weeks.

One reason for the different results for leptin and leptin receptor expression and serum leptin and insulin levels obtained from different studies may be due to the different species and models used. There are also differences even in the studies using the same species such as mice. It should be noted that the animal model used in the current study was mimicking the post-menopausal breast cancer mouse model, while most of the previous studies used either xenograft or chemically induced rodent models. Another difference between the studies is feeding of the animals. In the current study, food was removed from animals about five to seven hours before serum collection time while serum samples were collected after overnight fasting in most of the previous studies. 

## Conclusions

The present study is the first one to report protein expression levels of leptin and its receptor in a tissue other than mammary fat pad or MT tissues from MT-developed mouse model. Our data, together with data from previous studies, imply that leptin signaling may play more important roles in MT development locally at the mammary fat pad tissues rather than systemically. Further studies with local tissues where MT are developed are needed in order to clarify the role of leptin signaling in MT development in mouse models.
